# Prokaryotic and Eukaryotic Community Succession and Potential Parasitic Interactions During Two *Alexandrium pacificum* Blooms in Aotearoa New Zealand

**DOI:** 10.3390/toxins17090465

**Published:** 2025-09-17

**Authors:** Laura Biessy, Lincoln Mackenzie, Kirsty F. Smith

**Affiliations:** Molecular and Algal Ecology Group, Cawthron Institute, 98 Halifax St East, Nelson 7010, New Zealandkirsty.smith@cawthron.org.nz (K.F.S.)

**Keywords:** dinoflagellates, metabarcoding, depth sampling, Syndiniales, community composition

## Abstract

Harmful algal blooms (HABs), caused by the dinoflagellate *Alexandrium pacificum*, are increasingly frequent in the Marlborough Sounds, an important aquaculture region in Aotearoa New Zealand. *Alexandrium pacificum* produces paralytic shellfish toxins and blooms cause significant economic and ecological disruptions through contamination of edible shellfish. High-throughput sequencing of prokaryotic and eukaryotic communities was used to investigate community dynamics during bloom events across two consecutive summers. Distinct successional shifts were observed, with prokaryotic communities dominated by Rhodobacterales and Flavobacteriales during blooms, and increased abundance of the SAR11 clade (Pelagibacterales) post-bloom. Eukaryotic diversity was dominated by *Alexandrium* species (Gonyaulacales) during the bloom, and subsequently shifted towards Syndiniales, Gymnodiniales, and Peridiniales as blooms collapsed. Significant correlations indicated potential ecological roles for these taxa in bloom regulation, particularly Syndiniales, which could indicate parasitic interactions. Depth profiles revealed consistent microbial composition throughout the water column, validating depth-integrated sampling strategies for community studies. This research describes changes in the composition of microbial communities during two *A. pacificum* blooms, suggesting that species interactions (e.g., via parasitism) may play a role shaping bloom dynamics. Further studies incorporating environmental parameters, especially nutrient dynamics linked to anthropogenic activities, are necessary to better understand the drivers of blooms in this important aquaculture region.

## 1. Introduction

Over the past decades, reports of marine harmful algal blooms (HABs) have increased in frequency, duration, and geographical distribution. This has been attributed to global climate changes and to anthropogenic impacts, such as eutrophication [1,2]. HABs can cause significant impacts on human and animal health due to the production of toxic or bioactive compounds [3]. HABs can also have major economic [4,5] and environmental repercussions [6], for example, by restricting the harvest of shellfish until toxins depurate or by directly impacting on the health of fish and shellfish species [7].

In the marine environment, paralytic shellfish toxins (PSTs) are produced by HAB species from the genera *Alexandrium*, *Gymnodinium*, and *Pyrodinium*. PSTs negatively impact the shellfish aquaculture industry, causing harvest closures worldwide, with species from the genus *Alexandrium* the most commonly implicated [8]. *Alexandrium pacificum* (previously known as *A. catenella* in Aotearoa New Zealand) can develop long-lasting bloom events as well as extensive cyst beds, which can drive reoccurring bloom events. *Alexandrium pacificum* commonly occurs along the east coast of the North Island of Aotearoa New Zealand, but it was only detected for the first time in the top of the South Island in 2010. It now causes regular HAB events in one of the main shellfish production regions of Aotearoa New Zealand—the Marlborough Sounds [9]. Since 2010, it has resulted in widespread PST contamination of shellfish with associated closures of harvests for several months each year [9].

Conditions leading to the recurrence of HAB species have attracted a great deal of interest in the last two decades and previous studies have investigated the ecological niche of *A. pacificum* [10,11]. *Alexandrium pacificum* blooms were found to be associated with water surface temperature between 21 and 25 °C, salinities of around 34 psu or higher than 37 psu, and these blooms dominated when both NO_3_ and NH_4_ levels were high [12]. Increasingly, biotic interactions between microbial taxa, including competition, parasitism, and predation, have been recognized as critical factors regulating HABs dynamics. Previous research suggests certain bacterial groups may directly influence the growth and toxicity of dinoflagellate blooms, either by enhancing bloom initiation through nutrient cycling or by contributing to bloom termination via competition for essential nutrients [13,14,15]. Interspecific competition could also play a role in the promotion or decline of phytoplankton blooms via allelopathy [16], with some toxic *Alexandrium* species shown to have allelopathic activity [17]. It has also only been suggested that pressure from parasites can have a great impact on HAB dynamics, particularly their elimination [18].

High-throughput DNA sequencing techniques provide a powerful approach for capturing the diversity, composition, and succession patterns of microbial communities at high resolution, significantly expanding upon traditional microscopy and culture-based methodologies [19,20,21]. These methods enable a more comprehensive understanding of microbial interactions and their potential roles in influencing harmful algal bloom dynamics. Previous research utilizing high throughput sequencing for examining HAB interactions has largely focused on bacterial taxa and in freshwater environments [22,23]. However, a study on associations amongst bacteria, archaea, and microeukaryotes during a spring bloom of the dinoflagellate *A. pacificum* showed that Gammaproteobacteria and Bacteroidetes were predominant during the initial bloom stage, while Alphaproteobacteria, Cyanobacteria, and Actinobacteria were the most abundant taxa present during bloom onset and termination [24]. For eukaryotes, as expected, the bloom was dominated by *A. pacificum*, followed by a mixed assemblage of diatoms, green algae, rotifers, and other microzooplankton during bloom termination [24]. Interactions and response of microbial communities during marine HAB events are still not well understood.

The aim of this study was to investigate successional changes in both prokaryotic and eukaryotic communities during two distinct *Alexandrium pacificum* blooms in the Marlborough Sounds region of Aotearoa New Zealand, across two different summer periods, using high-throughput sequencing methodologies. The study site, Opua Bay, experiences regular blooms of *A. pacificum* (previously *A. catenella*). This species has been well characterized at this location and all strains are known to produce paralytic shellfish toxins [9,25]. During blooms, bivalve species exceed the regulatory level of 0.8 mg/kg shellfish meat as saxitoxin equivalents for extended periods of time [25]. Plankton dynamics in this region are critically understudied despite the recurrent economic and ecological impacts of HABs. By comparing microbial community structure in two distinct *A. pacificum* bloom events, the study provides insights into why certain microbial species dominate and identifies factors influencing bloom development and termination, thereby enhancing our ability to predict and manage these harmful events in this commercially important region of Aotearoa New Zealand.

## 2. Results

### 2.1. Microscopic Observations

**Figure 1:**
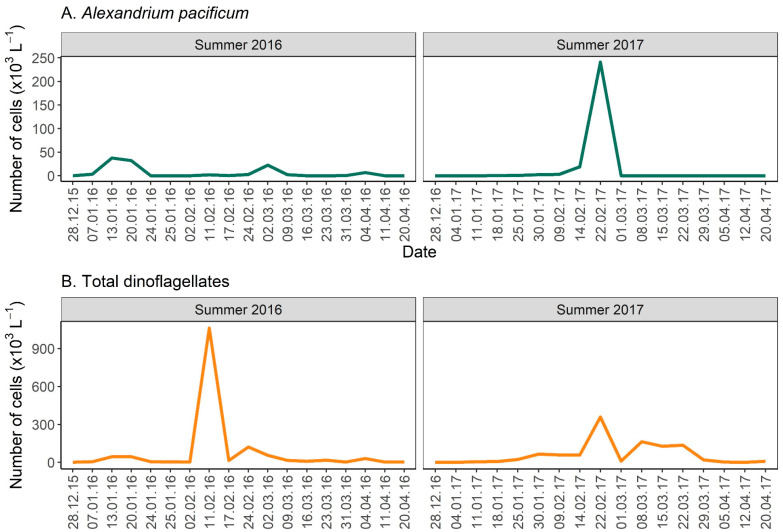
*Alexandrium pacificum* counts ((**A**); ×10^3^ cells L^−1^) and total dinoflagellates counts ((**B**); ×10^3^ cells L^−1^) using microscopic observations during algal blooms in Opua Bay, Aotearoa New Zealand in the summers of 2016 and 2017.

The microscopic observations showed three distinct bloom peaks of the dinoflagellate *A. pacificum* during the summer of 2016. The first and largest peak occurred on 7–24th of January, the second peak was from the 24th of February to 9th of March, and the smallest and last peak started on the 31st of March and finished on the 11th of April (Figure 1A). The total dinoflagellates number peaked on the 11th of February at 1062 × 10^3^ cells L^−1^, due to a mixed bloom of *Karenia longicanalis* (reaching 934 × 10^3^ cells L^−1^) and *Polykrikos geminatum* (reaching 126 × 10^3^ cells L^−1^; Figure 1B). During the 2017 summer, only one peak of *A. pacificum* occurred (9 February–1 March, Figure 1A), with extremely high cell concentrations (241 × 10^3^ cells L^−1^). This species dominated the total dinoflagellate counts for that date. This was followed by a mixed bloom of *Karenia longicanalis* and *Pentapharsodinium* sp.; both species do not produce PSTs (Figure 1B).

### 2.2. Community Composition Using Metabarcoding

After quality filtering the 65 water samples, the 16S rRNA gene barcode generated 3971 unique ASVs and the 18S rRNA barcode generated 2962 ASVs. There were no noticeable changes in the observed bacterial diversity during the *A. pacificum* blooms for both years with the observed number of ASVs varying between 100 and 330 but the eukaryotic diversity decreased during the blooms (from 200 to 300 ASVs to <50 ASVs during the blooms). Very few Metazoan ASVs were present in the samples (less than 20 at all times) and numbers did not fluctuate during the different blooms.

**Figure 2:**
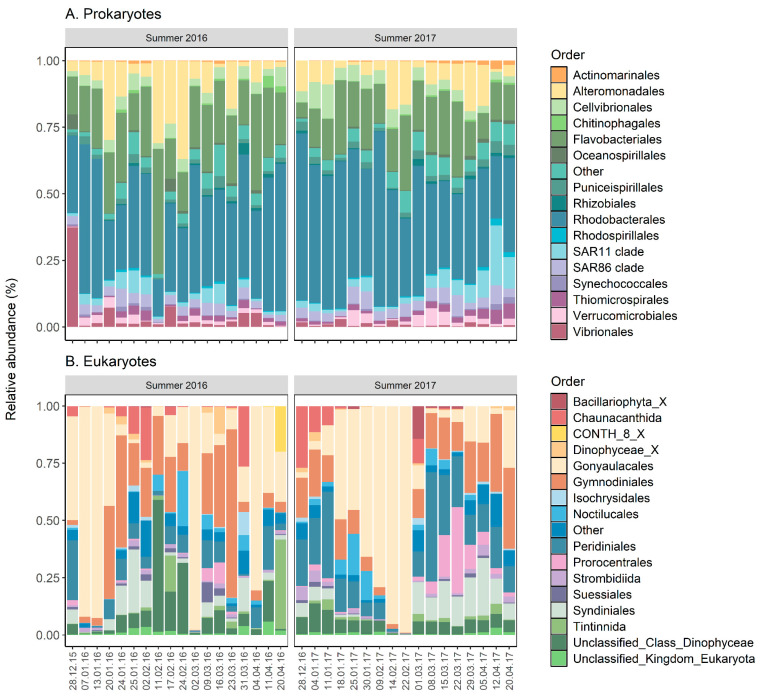
Stacked bar plots showing the relative abundance of prokaryotic (**A**) and eukaryotic (**B**) community composition at the order levels from water samples collected during blooms of *Alexandrium pacificum* in Opua Bay, Aotearoa New Zealand.

The two dominant orders of prokaryotes were Rhodobacterales (13–72%) and Flavobacteriales (4–51%) in all samples for both summers (Figure 2A). Alteromonadales were also present in most samples but at lower abundance (0–46%). There were no discernible changes in the prokaryotic composition during the blooms, except for an increase in the relative abundance of the SAR11 clade order following the *A. pacificum* bloom. Regarding the eukaryotic abundance, there were three distinct blooms of Gonyaulacales (with the genus *Alexandrium* representing 95–100% of the abundance) during the summer of 2016 and one single longer bloom during the summer of 2017. For both years, as the *Alexandrium* bloom disappeared, Syndiniales and Gymnodiniales abundances increased, as well as Peridiniales to a smaller extent (Figure 2B). Most of the families present in the Syndiniales order were either Amoebophyraceae or unclassified. Regarding Metazoan community composition, Crustacea was the dominant order (49–100%) in the water column for most samples and there were no changes observed during the blooms, with the dominant genera being from copepods (*Paracalanus* (27–90% of the total relative abundance), *Ointhona*, and *Centropages*).

**Figure 3:**
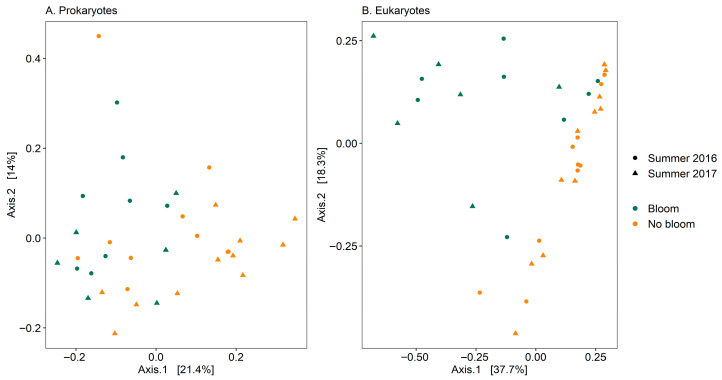
Principal Coordinates Analysis (PCoA) based on Bray–Curtis dissimilarities with 999 permutations of prokaryotic (**A**) and eukaryotic ((**B**); including metazoans) community composition of water samples collected during *Alexandrium pacificum* blooms and pre- and post-blooms (‘no bloom’) at the Amplicon Sequence Variant level from Opua Bay, Aotearoa New Zealand. For each axis, in square brackets, the percentage of variation explained was reported.

Multivariate analysis (PERMANOVA) showed significant differences in community composition amongst the two summers for prokaryotes (Bray–Curtis, *p* = 0.006, *F* = 2.41) but no significant difference in eukaryotes (Bray–Curtis, *p* = 0.37, *F* = 1.04) communities. After combining the communities from the two summers, the PERMANOVA analysis showed significant differences in community composition amongst the two stages of the blooms (during the bloom: ‘Bloom’, and pre- and post-bloom combined: ‘No bloom’; Figure 3) for prokaryotes (Bray–Curtis, *p* < 0.001, *F* = 2.90) and eukaryotes (Bray–Curtis, *p* < 0.001, *F* = 7.65).

**Figure 4:**
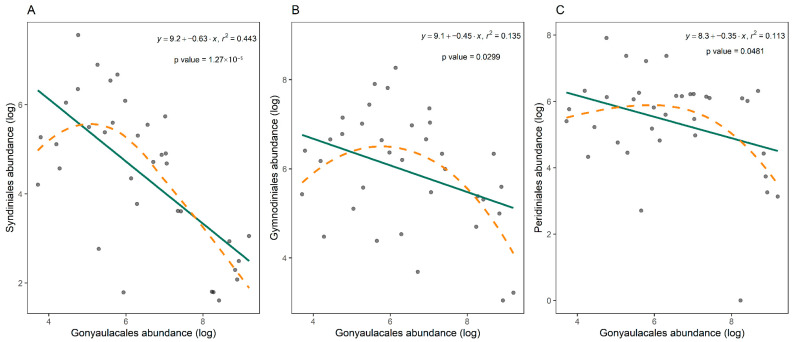
Linear regressions between (x) log-transformed Gonyaulacales abundance and (y) log-transformed abundances of Syndiniales (**A**), Gymnodiniales (**B**), and Peridiniales (**C**) from water samples collected during *Alexandrium pacificum* blooms. Correlation coefficients (r^2^), significance of the relationship (*p* < 0.05 taken as significant), and linear equations are presented. The solid line represents the linear regression, and the dashed line represents the smooth curve of a locally estimated scatterplot smoothing (loess) regression.

Three eukaryotic orders had significant relationships with changes in the abundance of Gonyaulacales (composed mainly by the genus *Alexandrium*). The strongest relationship was identified for Syndiniales (r^2^ = 0.443, *p* < 0.001, Figure 4A). There were also significant but weak linear relationships with Gymnodiniales (r^2^ = 0.135, *p* = 0.03; Figure 4B) and Peridiniales (r^2^ = 0.113, *p* = 0.048; Figure 4C).

### 2.3. Depth Profile and Community Composition

**Figure 5:**
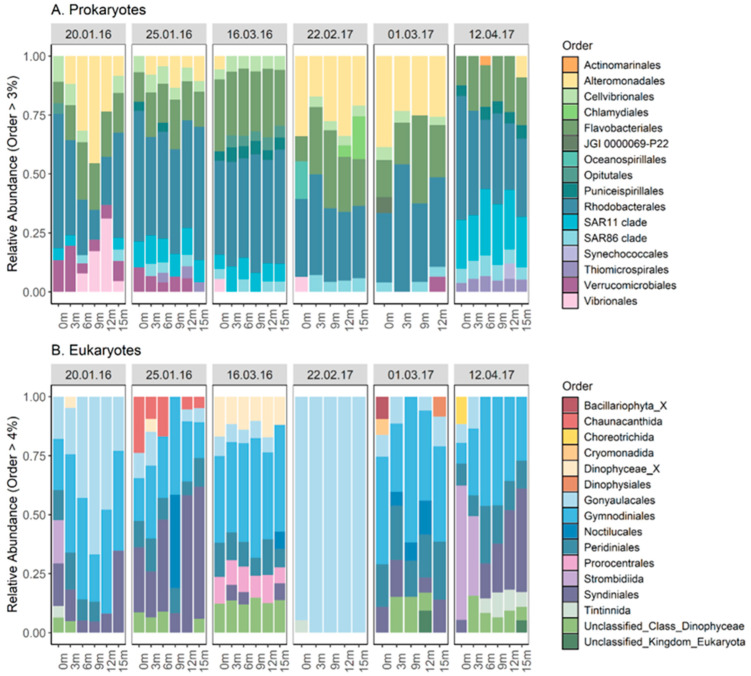
Stacked bar plots showing the relative abundance of prokaryotic (**A**) and eukaryotic (**B**) compositions at the order level in water samples collected at different depths in the water column in 2016 and 2017.

No noticeable changes were observed in the prokaryotic composition for each day at different depths, at the order level, with Rhodobacterales, Flavobacteriales, and Alteromonadales dominating the water column (Figure 5A). An increase in the relative abundance of bacteria from the SAR11 clade was observed once the bloom has terminated. There were also very little changes in the eukaryotic composition at different depths with Gonyaulacales dominating the entire water column during the bloom periods and Gymnodiniales being the dominant eukaryotic order outside of the bloom periods (Figure 5B). The order Strombidiida was only present in the 0 and 3 m samples and the Syndiniales abundance increased in the deeper samples (from 9 to 15 m). There were no changes in the metazoan composition for the different depths, with Crustacea almost exclusively dominating the water column with the copepod genus *Paracalanus*.

## 3. Discussion

The increasing frequency, duration, and geographic spread of HABs are of growing ecological and economic concern, including in Aotearoa New Zealand [5]. This study showed the recurrence of *A. pacificum* bloom events over two summers in the Marlborough Sounds region of Aotearoa New Zealand, a commercially important aquaculture area. This study focused on summer blooms from 2016 and 2017 but blooms of PST-producing *A. pacificum* still occur every year, or several times per year in Opua Bay, the study site. This region was and still is regularly affected by shellfish farm closures due to PST contamination [5,9]. The structure of prokaryotic and eukaryotic communities and bloom dynamics between the two sampled periods highlight the complexity of ecological factors that drive bloom initiation and collapse.

The shifts in prokaryotic and eukaryotic communities during the bloom succession underline the importance of microbial interactions as potential biological drivers. For instance, blooms of *Alexandrium pacificum* coincided with reduced microbial diversity and dominance of specific bacterial taxa such as Rhodobacterales and Flavobacteriales, and the succession towards Syndiniales and Gymnodiniales during bloom decline suggests these groups may play critical roles in the termination phase. Thus, understanding these microbial community dynamics and their interactions with environmental factors is vital to predict and potentially mitigate the detrimental impacts of recurrent *A. pacificum* blooms in this region.

One important shift in the prokaryotic community was the increase in the relative abundance of bacteria from the SAR11 clade order, now known Pelagibacterales, an order of the Alphaproteobacteria class [26]. This is one of the most abundant bacterial orders in ocean surface waters and Pelagibacterales are usually considered oligotrophs that can feed on dissolved organic carbon and nitrogen [27,28,29]. This result suggests the potential roles of Pelagibacterales in nutrient cycling during bloom collapse and should be investigated further.

Regarding the changes in the relative abundance of the order Gonyaulacales, identified as *Alexandrium pacificum* using microscopy, increases in the relative abundance of Syndiniales, Gymnodiniales, and Peridiniales taxa were observed after the collapse of the blooms. These findings indicate that some of these groups could have roles in the ecology of the *Alexandrium* blooms, such as parasitism, competition, allelopathy, or grazing pressure. This was particularly noticeable, with a strong significant and positive relationship observed for the group Syndiniales, with a low relative abundance before the bloom and an increase after the bloom. Syndiniales (Dinophyceae) are a common and diverse parasitic group, but their ecology remains poorly understood [30]. All described Syndiniales automatically kill their host, including other eukaryotes (e.g., dinoflagellates) and metazoans [31]. This finding suggests a parasitic relationship potentially influencing bloom termination between Gonyaulacales (*A. pacificum* in this case) and Syndiniales. A previous study hypothesized that the decline in toxic *Alexandrium minutum* bloom in a French estuary may have been due to an introduced dinoflagellate parasite (e.g., *Amoebophyra* sp.) [18].

Our study also showed that copepods (*Paracalanus* spp.) dominated the water column both during the blooming period and outside of blooming events. Previous studies have looked at the importance of mesozooplankton (i.e., copepods) grazing as a potential factor of HAB collapse. Results showed that copepods can graze on toxic microalgae, regardless of the toxicity levels [32]; the ingestion of toxic dinoflagellate cells is species-specific [33] and could be an important factor affecting the bloom development of the dinoflagellates [34]. This highlights the potential of copepods, particularly species of *Paracalanus*, as an avenue for exploring HAB mitigation in Aotearoa New Zealand.

Metabarcoding techniques have enabled the study of fine-scale HAB dynamics, with the ability to undertake a detailed, culture-free analysis of microbial communities throughout the water column during a bloom event, characterizing taxa from multiple trophic levels. But there are also limitations to this method. One crucial step in metabarcoding studies is the choice of a targeted gene region, as it could affect the community composition obtained [35,36,37]. For the eukaryotic18S rRNA gene, the short V9 region or the longer V4 region is the most commonly targeted and thus well represented in reference databases [38,39]. However, dinoflagellates have unusually large genomes with associated high rDNA copy number and lots of intra-specific variation [40,41]. It is worth noting that metabarcoding studies of phytoplankton species can be biased towards dinoflagellates and this may influence the levels of diversity and relative abundance of these taxa in studies that target rRNA genes [42]. Additionally, when studying community dynamics, the taxonomic identification of species based on DNA sequence data relies heavily on accurate and well-curated reference databases. Regarding the prokaryotic communities, articles assigned to cyanobacteria were low in this study, but it is worth noting that the primers used can be biased towards detection of general bacterial and might not be appropriate for the detection of cyanobacterial species [43]. Also, the reference databases for all eukaryotes, particularly for dinoflagellates, vary in completeness and availability, depending on the taxonomic group targeted [44]. Relevant to this study, very little is known about the majority of Syndiniales species [45], and this could limit their identification from eukaryotic communities.

The differences in both prokaryotic and eukaryotic community structure at different depths in the water column were also studied. For each sampling date, only some small changes in the relative abundance of the communities within the water column were observed in the prokaryotic communities. It was similar for the eukaryotic communities, apart from the order Strombidiida which was only present on the top layer (0–3 m), and the order Noctilucales, only present in the middle layer (3–12 m). This highlights that a composite sample of the whole water column, when possible, is enough to have a representative community composition for the entire water column for environmental DNA studies, as long as replication is adequate.

Environmental conditions and parameters were not recorded in this study, limiting the understanding of abiotic drivers of *A. pacificum* blooms in the Marlborough Sounds region and this should be the focus of future studies. This information would help understand if *A. pacificum* blooms in this region are also associated with a specific range of water surface temperatures (21–25 °C), salinities (34–37 psu), and if these blooms dominate when both NO_3_ and NH_4_ levels were high, as demonstrated by Bravo, Vila, Masó, Figueroa, and Ramilo [12]. Blooms of *A. pacificum* have been a recurring issue in the Marlborough Sounds since 2010, particularly in Opua Bay, the focus of this study [9,46]. The Tory channel, where the study site is located, is naturally high in nitrate due to upwelling from the nearby Cook Strait, a strait that separates the North and South Islands of Aotearoa New Zealand [47], and it is likely that these conditions promote the formation of HABs. Prior to European settlement, the shoreline of Opua Bay was covered in lowland forest and dominated by native forest in the higher elevations [48]. Native forests were cleared in the mid-1800s and today, the majority of the catchment is colonized by introduced pine trees *Pinus radiata* [49]. Impacts of forestry on the aquatic ecosystems can be significant (e.g., increase in fine sediments and nutrients after logging activities [49,50,51,52]) and the impacts of forestry practices on the ecology of algal communities and water quality has been demonstrated in freshwater systems [53,54,55]. Potentially, changes in the catchment from the pine forestry in Opua Bay could be associated with the exacerbation of HAB species in this region. Environmental conditions preceding bloom events should be prioritized for any future studies to determine abiotic drivers of HABs.

## 4. Conclusions

To conclude, this study provided insights into microbial community dynamics associated with *A. pacificum* blooms in an important aquaculture region. We showed that prokaryotic communities were dominated by Rhodobacterales and Flavobacteriales during blooms, with Pelagibacterales (SAR11 clade) increasing post-bloom, suggesting a role in nutrient cycling during bloom decline. Eukaryotic succession was characterized by *Alexandrium* dominance followed by rises in Syndiniales, Gymnodiniales, and Peridiniales, with Syndiniales strongly correlated with bloom collapse, pointing to potential parasitic interactions. Depth-integrated sampling was validated as representative of whole water column dynamics, strengthening its use for future HAB studies. These findings highlight the novel contribution of combining high-throughput sequencing with bloom ecology to reveal biotic interactions that may regulate bloom termination. They will help focus efforts for future investigations into microbial interactions and environmental and ecological drivers, critical for developing predictive tools and targeted management strategies to mitigate HABs in Aotearoa New Zealand. Future research should prioritize characterizing environmental factors, including nutrient dynamics influenced by nearby forestry and aquaculture practices, to further understand the conditions triggering HAB events.

## 5. Materials and Methods

### 5.1. Study Site

**Figure 6:**
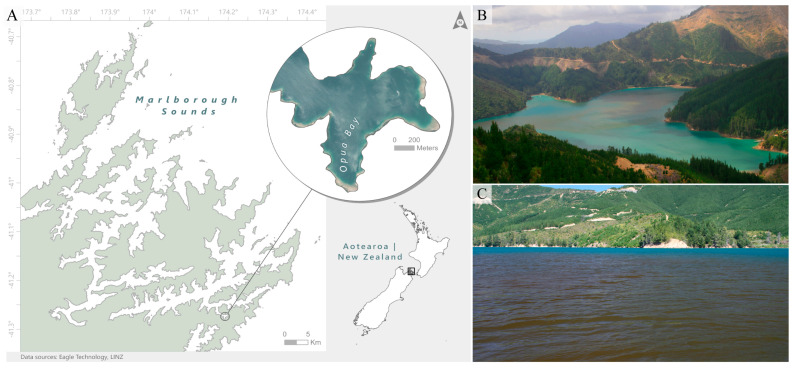
(**A**) Map of the study location, Opua Bay (Marlborough Sounds, Aotearoa New Zealand), and photographs from January 2016 of *Alexandrium pacificum* blooms within Opua Bay, showing (**B**) aerial view of the Bay when a bloom is occurring, and (**C**) water discoloration.

Samples were collected from a boat moored at a sampling station in Opua Bay (Marlborough Sounds, Aotearoa New Zealand; 41°16′23.2″ S 174°12′04.1″ E; Figure 6A). This site was chosen as it has recurring dense blooms of PST-producing *A. pacificum* every year since 2010 [9] and is known to contain particularly high numbers of *A. pacificum* cysts in the sediments [46]. Sampling was carried out regularly over two summer periods (December 2015 to April 2016 and December 2017 to April 2018). Water samples were collected using van Dorn water bottle casts, at three-meter intervals from surface to 15 m (i.e., at 0 m, 3 m, 6 m, 9 m, 12 m, and 15 m). Depth samples were then pooled and subsampled for analysis, as described below. To understand the differences in communities at different depths, during the two summers, some sampling points were randomly selected and also analyzed as discrete depth profiles. A CTD/fluorometer cast to measure chlorophyll-*a* fluorescence was also made at each sampling occurrence.

### 5.2. Microscopy Analysis

From each sample, a 100 mL subsample was preserved in Lugol’s solution for microscopic analyses. Subsamples (10 mL) were taken and examined using inverted light microscope (LM) and Utermöhl chambers. The number of dinoflagellate cells per liter, including *A. pacificum*, was calculated.

### 5.3. DNA Extraction, Polymerase Chain Reaction, High Throughput Sequencing, and Bioinformatics

From each water sample, two liters were filtered on a sterile 0.45 µm filter and DNA was extracted from the filter using the DNeasy PowerSoil Kit (Qiagen, Hilden, Germany), following the manufacturer’s instructions, and negative extraction controls were included every 24th sample. The V3-V4 regions of the bacterial 16S ribosomal RNA (rRNA) gene (Bact341F- 5′- CCT ACG GGN GGC WGC AG-3′ and Bact785R- 5′-GAC TAC HVG GGT ATC TAA TCC-3′) [56,57] and the V4 region of the eukaryote nuclear 18S rRNA gene (Uni18SF: 5′-AGG GCA AKY CTG GTG CCA GC-3′ and Uni18SR: 5′-GRC GGT ATC TRA TCG YCT T-3′) [58] were amplified by Polymerase Chain Reaction (PCR). PCRs, purification, and library preparation were undertaken, as described in Biessy et al. [59], and using a Nextera Index kit (Illumina, San Diego, CA, USA).

Bioinformatic pipelines for both the 16S and 18S rRNA genes were identical unless otherwise stated. Primers were removed from the raw reads using ‘cutadapt’ with 1 mismatch allowed [60] and subsequently processed using the DADA2 package [61] within R [62]. Reads were truncated (230 and 228 bp for forward and reverse reads, respectively) and filtered with a maximum number of ‘expected errors’ (maxEE) threshold of two (forward reads) and four (reverse reads). Those reads not matching this criterion were discarded from further analysis. A parametric error matrix was constructed based on the first 108 bp of the sequences. The processes for dereplication, singletons, and chimera removal were undertaken using the protocol described in Biessy et al. [63].

The resulting chimera-checked Amplicon Sequence Variants (ASVs) were used for taxonomic classification against the SILVA 138 database for the 16rRNA [64] and the PR2 database [65]. The sequences were classified based on the rdp classifier [66] with a bootstrap of 50 so as to be able to obtain classifications at higher taxonomic levels. The results were combined into a phyloseq object [67] and for the 16S rRNA dataset sequences assigned as microeukaryotes, chloroplasts and mitochondria were removed. The sums of the reads from the negative controls were evaluated and contamination ASVs were subtracted from all samples.

For comparisons between samples, subsampling to an even depth was undertaken for each sample at a depth of 9000 and 10,000 reads for the prokaryotic and eukaryotic datasets, respectively. The Metazoan were a subset out of the eukaryotic sequences to be analyzed on their own. Alpha diversity numbers were calculated using the estimate_richness function from the phyloseq package in R [67] at any given day; however, statistical differences in alpha diversity could not be assessed due to lack of replication. Phylogenetically annotated 16S rRNA and 18S rRNA sequences were used to characterize prokaryotic and eukaryotic community compositions of each island type at order, family, and genus levels. Stacked bar plots, and abundance bubble plots were generated using the package ggplot2 [68] in R, based on the average relative abundance of sequence reads attributed to a given order, family, or genus within each summer. The proportion of explained variance between each year on the ASV composition dissimilarities was evaluated using a permutational analysis of variance (PERMANOVA) test implemented by the adonis function in the vegan package [69]. The PERMANOVA test was also used to determine the dissimilarities between the community composition during the bloom (>25% relative abundance of the genus *Alexandrium* at any given day) and pre- or post-bloom and visualization of the observed patterns was obtained by means of a principal coordinates analysis (PCoA) using a Bray–Curtis similarity matrix. Relationships between some eukaryotic and prokaryotic orders and the Gonyaulacales order were determined using linear regressions.

## Data Availability

Sequence Read Archive Submission number SUB15545316.
